# The dual timescales of gait adaptation: initial stability adjustments followed by subsequent energetic cost adjustments

**DOI:** 10.1242/jeb.249217

**Published:** 2024-11-28

**Authors:** Sarah A. Brinkerhoff, Natalia Sánchez, Meral N. Culver, William M. Murrah, Austin T. Robinson, J. Danielle McCullough, Matthew W. Miller, Jaimie A. Roper

**Affiliations:** ^1^ Department of Neurology, University of Alabama at Birmingham, Birmingham, AL 35233, USA; ^2^ Department of Physical Therapy, Chapman University, Irvine, CA 92618, USA; ^3^ Fowler School of Engineering, Chapman University, Orange, CA 92866, USA; ^4^ School of Kinesiology, Auburn University, Auburn, AL 36849, USA; ^5^ Department of Kinesiology, Department of Educational Foundations, Leadership, and Technology, Auburn University, Auburn, AL 36849, USA; ^6^ Department of Kinesiology, Indiana University Bloomington, Bloomington, IN 47405-7109, USA; ^7^ Edward Via College of Osteopathic Medicine, Auburn Campus, Auburn, AL 36832, USA

**Keywords:** Gait mechanics, Motor adaptation, Locomotor learning, Motor learning

## Abstract

Gait adaptation during bipedal walking allows people to adjust their walking patterns to maintain balance, avoid obstacles and avoid injury. Adaptation involves complex processes that function to maintain stability and reduce energy expenditure. However, the processes that influence walking patterns during different points in the adaptation period remain to be investigated. We assessed split-belt adaptation in 17 young adults aged 19–35. We also assessed individual aerobic capacity to understand how aerobic capacity influences adaptation. We analyzed step lengths, step length asymmetry (SLA), mediolateral margins of stability, positive, negative and net mechanical work rates, as well as metabolic rate during adaptation. Dual-rate exponential mixed-effects regressions estimated the adaptation of each measure over two timescales; results indicate that mediolateral stability adapts over a single timescale in under 1 min, whereas mechanical work rates, metabolic rate, step lengths and SLA adapt over two distinct timescales (3.5–11.2 min). We then regressed mediolateral margins of stability, net mechanical work rate and metabolic rate on SLA during early and late adaptation phases to determine whether stability drives early adaptation and energetic cost drives late adaptation. Stability predicted SLA during the initial rapid onset of adaptation, and mechanical work rate predicted SLA during the latter part of adaptation. Findings suggest that stability optimization may contribute to early gait changes and that mechanical work contributes to later changes during adaptation. A final sub-analysis showed that aerobic capacity levels <36 and >43 ml kg^−1^ min^−1^ resulted in greater SLA adaptation, underscoring the metabolic influences on gait adaptation. This study illuminates the complex interplay between biomechanical and metabolic factors in gait adaptation, shedding light on fundamental mechanisms underlying human locomotion.

## Teaser

**Highlighted Article:** The complex dynamics of human gait adaptation highlight rapid stability optimization and gradual adjustments in metabolic rate and step kinetics and kinematics, offering insights into energy-saving mechanisms.

## INTRODUCTION

Adjusting gait to meet environmental demands is a critical and inherent feature of bipedal walking, thought to be accomplished through trial-and-error adaptation of typical walking patterns (Martin et al., 1996). In laboratory settings, gait adaptation can be assessed via split-belt treadmill walking, consisting of a treadmill with independently controlled belts moving at different speeds. Gait adaptation in response to treadmill walking is robust and observable through various kinematic and kinetic measures, including alterations in step lengths (Reisman et al., 2005; Sánchez et al., 2021), mediolateral margin of stability (Buurke et al., 2018) and mechanical work done by the legs (Sánchez et al., 2019, 2021; Selgrade et al., 2017). Although these adaptive responses to split-belt perturbations have been consistently shown experimentally, the process(es) that drive these responses remain unknown.

Prior work has postulated that gait adaptation during split-belt treadmill walking is driven by error-based learning, where interlimb kinematic asymmetry serves as an error signal that the nervous system attempts to minimize over the course of adaptation (Malone et al., 2012; Reisman et al., 2005). Although interlimb asymmetry does adapt, recent work has proposed a use-dependent learning framework (Wood et al., 2021) and has demonstrated that, with sufficient time and practice under novel walking conditions, people tend to adopt asymmetric gait patterns (Sánchez et al., 2021; Stenum and Choi, 2020) that reduce the energetic costs of walking (Finley et al., 2013; Sánchez et al., 2019, 2021). Additionally, the acquisition of a novel gait pattern can be influenced by reinforcement learning, where specific and binary kinematic feedback can drive the learning process (Wood et al., 2024). Recent simulation studies have demonstrated that gait adaptation in response to a continuous perturbation is driven first by modifying stability (Seethapathi et al., 2024) and then by reducing metabolic cost (Price et al., 2023; Seethapathi et al., 2024). The hypothesis that early adaptation functions to maintain stability is supported by recent data demonstrating reduced early gait perturbation (i.e. within the first five strides) when participants are allowed to hold on to handrails (Park and Finley, 2022). This early phase of gait adaptation seems to correspond to the first of two timescales of adaptation in step length asymmetry (SLA) during continuous gait adaptation (Darmohray et al., 2019; Mawase et al., 2013; Roemmich et al., 2016; Sánchez et al., 2021). The second timescale may correspond to metabolic cost adaptation (Price et al., 2023; Seethapathi et al., 2021) and is supported by work suggesting that gait adaptation typically leads to asymmetric gait patterns if they result in reduced energetic cost (Sánchez et al., 2021; Stenum and Choi, 2020). The stability and energetic aspects of adaptation can be measured experimentally using mediolateral margin of stability (ML MoS) as a proxy for mediolateral stability (Buurke et al., 2018), using metabolic rate from indirect calorimetry as a proxy for metabolic cost (Buurke et al., 2018; Finley et al., 2013; Roemmich et al., 2019; Roper et al., 2013; Sánchez et al., 2019, 2021), and mechanical work by the legs as a proxy for mechanical cost (Buurke et al., 2018; Finley et al., 2013; Sánchez et al., 2019, 2021; Selgrade et al., 2017). Thus, we can objectively assess the underlying processes proposed to drive locomotor adaptation (Price et al., 2023; Seethapathi et al., 2021).

Although our group and others have independently investigated kinematics, stability, metabolic rate and mechanical work by the legs during gait adaptation (Brinkerhoff et al., 2023, 2021; Buurke et al., 2018; Darter et al., 2018; Finley et al., 2013; Park and Finley, 2022; Roemmich et al., 2019; Roper et al., 2013, 2017, 2019; Sánchez et al., 2019, 2021), the field has yet to explore the timescales of each of these processes and the relationships between them. Thus, the primary purpose of the present study was to comprehensively investigate the dynamics of gait adaptation by examining the interplay between stability, metabolic cost and mechanical cost. Specifically, we aimed to unravel the intricate relationships and timescales associated with kinematic measures of adaptation (SLA, fast-leg step length and slow-leg step length); measures of stability (ML MoS of the fast leg and ML MoS of the slow leg); and energetic measures, such as metabolic rate and work done by the legs (net mechanical work rate, positive fast-leg mechanical work rate, negative fast-leg mechanical work rate, positive slow-leg mechanical work rate and negative slow-leg mechanical work rate). We hypothesized two distinct timescales of adaptation: (1) ML MoS, as a proxy for stability, would adapt quickly (Park and Finley, 2022; Seethapathi et al., 2021), whereas (2) mechanical and metabolic work rates would adapt slowly (Darmohray et al., 2019; Mawase et al., 2013; Roemmich et al., 2016; Sánchez et al., 2021; Seethapathi et al., 2021). We also sought to define the relationships between adaptation and the processes of adaptation (ML MoS, metabolic rate and net mechanical work rate done by the legs). We hypothesized that ML MoS would predict initial but not later SLA, whereas mechanical work rate and metabolic cost would predict later but not initial SLA. Finally, to describe the role of metabolic capacity on gait adaptation, we regressed maximal oxygen consumption (*V*_O_2__ peak) from a graded exercise test on SLA and metabolic rate adaptations. This work will help inform the field on the objectives that drive gait adaptation in younger adults, which can provide nuanced insight to inform biomechanical models of gait, guide clinical decision-making and gait rehabilitation strategies, and develop and/or refine assistive gait technologies.

## MATERIALS AND METHODS

### Participants

To determine sample size, we simulated samples of sizes *N=*6–30 from known parameters (determined from pilot data) for SLA, metabolic rate and ΔML MoS of the fast leg. We compared the model estimates from each simulated sample with the actual model parameters. We used reasonable heuristics to determine the minimum sample size that resulted in stable estimates. Specifically, we identified the sample size *N* at which the standard deviation of the differences between the simulated model growth estimates (*r*_f_ and *r*_s_ in Eqn 3) and the true estimates, from samples of size *N*−30, was less than or equal to half the standard deviation of the differences observed in the full sample size range before *N*. This approach allowed us to identify the point at which the variability in our growth estimates stabilized. The minimum sample sizes to achieve stable estimates were determined for SLA (*N*=17), metabolic rate (*N*=14) and ΔML MoS (*N*=16) of the fast leg.

We recruited *n*=17 participants from a convenience sample of young adults aged 19–35 for participation in this study (Table 1). Participants were excluded if they reported cardiovascular, pulmonary, renal, metabolic, vestibular or neurologic disorders; any lower-extremity injuries or surgeries in the past 12 months; or a prior anterior cruciate ligament injury. Participants completed written informed consent via Qualtrics prior to visiting the laboratory for participation. The Auburn University Institutional Review Board approved all procedures.

**Table 1.**
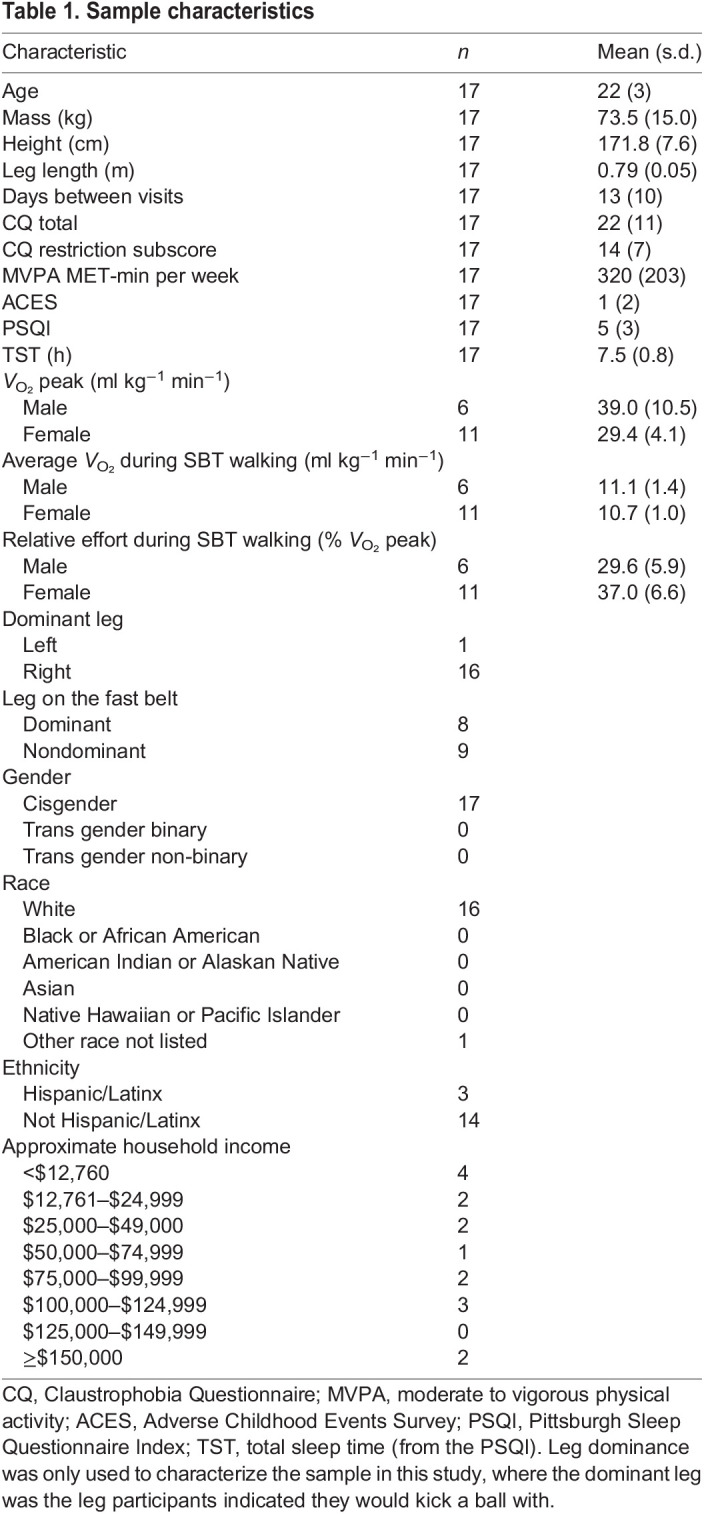
Sample characteristics

### Experimental protocol

Participants completed demographic intake questions and four surveys via Qualtrics before visiting the laboratory. Participants completed the Godin Leisure-Time Exercise Questionnaire (Godin and Shephard, 1985) to estimate their weekly exercise over the past 3 months. Participants were excluded if they performed more than 750 MET-minutes (metabolic equivalent of task, where 1 MET is the amount of energy spent at rest) per week of moderate–vigorous exercise (equivalent to 150 min of moderate exercise per week) to avoid the confounding effect of exercise on adaptation (Brinkerhoff et al., 2023). Participants also completed the Claustrophobia Questionnaire (Radomsky et al., 2001). Participants were excluded if they reported a claustrophobia score greater than 48 or a restriction subscore greater than 32 (Radomsky et al., 2001) to prevent adverse events resulting from wearing the metabolic face mask. To describe the sample, participants completed the Adverse Childhood Experiences questionnaire which is scored 0 (better) to 10 (worse) (Felitti et al., 1998), the Pittsburgh Sleep Quality Index which is scored 0 (better) to 21 (worse) (Buysse et al., 1989), and questions about socioeconomic status, race, ethnicity, sex assigned at birth, gender identity and leg dominance (where the dominant leg is the leg that they indicate they would kick a ball with).

Participants visited the lab twice. During visit 1, participants completed a treadmill graded exercise test to volitional exhaustion (BSU/Bruce Ramp protocol; Kaminsky and Whaley, 1998). Maximal oxygen consumption (i.e. *V*_O_2_ _peak) was measured using indirect calorimetry (Parvomedics TrueOne2400, Sandy, UT, USA) and was used to characterize aerobic capacity for our sample. Criteria for attainment of *V*_O_2__ peak included achieving at least two of the following: a respiratory exchange ratio (RER) greater than 1.1, a rating of perceived exertion (RPE) greater than 17 (6 to 20 scale), a heart rate within 10 beats min^−1^ of their age-predicted maximum, and a plateau in O_2_ with increasing exercise intensity (American College of Sports Medicine, 2019).

During visit 2 (scheduled within 3 months of the graded exercise test), participants completed the gait adaptation protocol on a split-belt treadmill (SBT) (Prokop et al., 1995; Reisman et al., 2005). Metabolic rate was measured breath-by-breath with indirect calorimetry (Parvomedics TrueOne2400). Kinematic data were recorded from reflective markers placed bilaterally on the anterior superior iliac spine and the lateral malleoli of the ankles using a 17-camera motion capture system (VICON, Vicon Motion Systems Ltd, Oxford, UK).

**Fig. 1.**
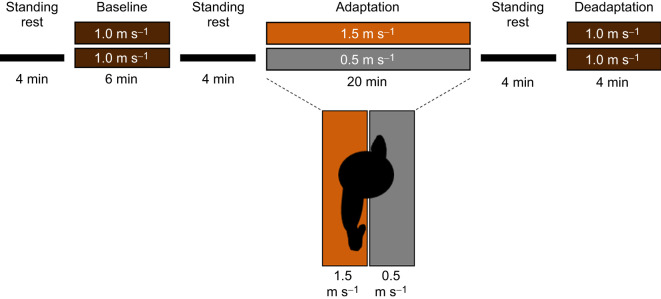
**Experimental protocol.** Orange, fast leg; gray, slow leg. Participants stood for 4 min, followed by a 6-min walk at 1.0 m s^−1^ (baseline). We measured each participant's average step lengths during the last five strides of baseline to determine which leg naturally took a longer step when treadmill walking, and which leg naturally took a shorter step. After a 4-min break, participants walked for 20 min with the belt under the shorter-stepping leg moving at 1.5 m s^−1^, and the belt under the longer-stepping leg moving at 0.5 m s^−1^ (adaptation). After another 4-min break, participants walked for 4 min with the belts tied at 1.0 m s^−1^ (deadaptation).

The experimental protocol is shown in Fig. 1. Participants were instructed not to use the treadmill handrails for the duration of treadmill walking. First, participants stood for 4 min to obtain their standing metabolic rate. Then, they walked for 6 min at 1.0 m s^−1^ (baseline), followed by at least 4 min of standing or until the participant's metabolic rate returned to standing baseline levels. We measured each participants' average step lengths during the last five strides of baseline to determine which leg naturally took a longer step when treadmill walking, and which leg naturally took a shorter step. Participants then walked for 20 min with the belt speeds split, such that the belt under the leg that took a shorter step during tied baseline walking moved at 1.5 m s^−1^, and the belt under the leg that took a longer step during tied baseline walking moved at 0.5 m s^−1^ (‘adaptation’). Following adaptation, participants stood for at least 4 min or until their metabolic rate returned to standing metabolic rate. Finally, they walked for 4 min with the belts tied at 1.0 m s^−1^ (‘deadaptation’).

### Data analysis

Kinematic data were recorded at 100 Hz and were lowpass filtered with a fourth-order Butterworth filter with a cut-off frequency of 6 Hz. Ground reaction force (GRF) data were obtained for each leg using an instrumented SBT, recorded at 1000 Hz from two separate force plates, and lowpass filtered with a fourth-order zero-phase Butterworth filter with a cut-off frequency of 20 Hz.

#### Step length and step length asymmetry

Step length was calculated for each leg as the distance between the ankle markers along the anterior–posterior walking axis at the ipsilateral foot strike. SLA was calculated and normalized to stride length (Eqn 1):
(1)

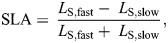
where *L*_S,fast_ is the step length when the leg on the fast belt strikes the belt, and *L*_S,slow_ is the step length when the leg on the slow belt strikes the belt. A negative SLA indicates that the leg on the slow belt is taking a longer step than the leg on the fast belt, and an SLA of zero indicates that the legs are taking steps of equal length.

#### Mediolateral margin of stability

We calculated ML MoS at every frame as the distance (cm) in the frontal plane between the extrapolated center of mass (CoM) and the base of support, as calculated by Buurke and colleagues (Buurke et al., 2018; Hof et al., 2005). Methods in-brief are provided. Data from the two force plates were combined to simulate a single plate for GRF and for ML center of pressure (CoP). ML CoP was calculated from forces and moments. ML CoM acceleration was calculated by dividing ML GRF by body mass and was twice-integrated to obtain a ML CoM relative position. Summing ML CoM relative position and ML CoP determined the ML CoM absolute position (Schepers et al., 2009). The velocity of this ML CoM absolute position was used to calculate extrapolated center of mass (xCoM) to find ML MoS (Hof et al., 2005). Fast-leg ML MoS was defined as the ML MoS at slow-leg foot-off, and slow-leg ML MoS was defined as the ML MoS at fast-leg foot-off. We chose to assess ML MoS at contralateral toe-off because this is the point in the gait cycle when the CoM transitions from one limb to the other, which is suggested to have the highest metabolic demand and potentially the lowest stability (Kuo et al., 2005). An ML MoS of zero would indicate the xCoM was directly over the CoP; negative values would indicate that the xCoM was more lateral than the CoP and the person would have to take a corrective step; and a positive ML MoS would indicate that the xCoM was more medial than the CoP. During typical walking, ML MoS is most often positive, where the larger a positive value, the further that the xCoM was from crossing over the CoP, and therefore connoted a more stable position (Hof et al., 2005).

#### Metabolic rate

We calculated gross energy expenditure (*E*_gross_; J min^−1^) for every breath (Garby and Astrup, 1987):
(2)

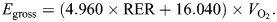
Metabolic rate was calculated by dividing energy expenditure by 60 s (J s^−1^, *W*). Net metabolic rate was calculated by subtracting standing baseline energy (*W*_net_). Relative net metabolic rate was calculated by dividing net metabolic rate by the participant's mass in kg (*W*_net_ kg^−1^; Eqn 2).

On average across 20 min of SBT walking adaptation, participants were working at a relative effort of 34.4% (s.d.=7.1%) of their *V*_O_2__ peaks (Table 1).

#### Mechanical work rates

We calculated positive and negative mechanical work generated by the legs with a custom MATLAB program that used a point-mass model to estimate mechanical work generated by the legs on the treadmill and on the CoM (Sánchez et al., 2019, 2021; Selgrade et al., 2017). Force data were partitioned by strides and the CoM accelerations in each direction were time-integrated to obtain the CoM velocity. The instantaneous power of each leg was obtained by summing the power generated by the leg on the body and the power generated by the leg on the treadmill; we added the dot product of the CoM velocity and the GRF (power generated by the leg on the body) to the dot product of the belt velocity and force applied to the belt, which is the inverse of the GRF (power generated by the leg on the treadmill). Positive and negative work done by the leg was obtained by separately calculating the time integral of the positive and negative instantaneous power over the stride. Finally, work done by the leg was transformed into mechanical work rate by dividing the positive and negative work by the stride duration to account for changing stride duration during adaptation.

### Statistical analyses

All statistical analyses were conducted in R (https://www.r-project.org/). Data are reported as means±s.e.m. Because metabolic data were obtained on a per-second basis, we also measured gait data by time rather than steps to allow comparison of timescales across the different variables. Mixed-effects nonlinear regression models were fit to each measure with the ‘nlme’ package (https://CRAN.R-project.org/package=nlme). All models were fitted with a maximum likelihood estimation, and all models contained a random effect such that the outcome measure's estimated plateau was allowed to vary by participant.

We first determined the timescale (in seconds) of adaptation of each of the 11 measures: (1) SLA, (2) fast-leg step length, (3) slow-leg step length, (4) ML MoS of the fast leg, (5) ML MoS of the slow leg, (6) metabolic rate, (7) net mechanical work rate done by the legs, (8) positive fast-leg mechanical work rate, (9) negative fast-leg mechanical work rate, (10) positive slow-leg mechanical work rate and (11) negative slow-leg mechanical work rate. We removed the data from the initial transient increase in metabolic rate owing to the onset of exercise, to only estimate the adaptation of metabolic rate after exercise onset.

We fit dual-rate exponential models to each measure (Eqn 3), estimating the adaptation of each measure over two distinct timescales:
(3)

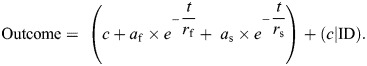
In the two-exponent models, *c* was the estimated plateau if seconds (*t*) went to infinity; *a*_f_ was the initial value of the fast timescale of adaptation; *r*_f_ was the growth rate of the fast timescale of adaptation; *a*_s_ was the initial value of the slow timescale of adaptation; *r*_s_ was the growth rate of the slow timescale of adaptation. For each measure, we confirmed that a two-exponential model fit the data better than a one-exponential model using AIC.

We also binned each measure by ‘initial’ and ‘later’ adaptation phases. Initial adaptation included the biomechanic data from the start of adaptation until the time (*r*_f_) when the fast timescale of adaptation reached 63.2% of its final value, with metabolic data lagged 3 min behind to account for the time required for oxygen consumption and carbon dioxide production to reach a steady state (Sánchez et al., 2019; Selinger and Donelan, 2014). Later adaptation included biomechanic data from minutes 15 to 17 and metabolic data from minutes 18 to 20. Each measure was then averaged over 3-s bins during each phase.

To test the hypothesis that stability drives initial stages of adaptation, and that energetic cost adaptation drives later stages of adaptation, linear mixed-effects models were used to analyze the interactions of epoch (initial SLA versus later SLA adaptation) with fast- and slow-leg ML MoS and net mechanical work on SLA. A mixed-effects model also analyzed the interaction of epoch and SLA on the metabolic rate, which was lagged behind SLA by 3 min.

Finally, as a sub-analysis to follow up on a prior study (Brinkerhoff et al., 2023), we analyzed the relationships between aerobic capacity and gait adaptation. To this end, the changes in SLA and metabolic rate at the end of adaptation were calculated. SLA and metabolic rate were each averaged over initial adaptation (0 to *r­*_f_ seconds) and the last 2 min of adaptation, and then initial adaptation values were subtracted from late adaptation values. Linear OLS regressions estimated the effect of gender-centered *V*_O_2__ peak on SLA adaptation and on metabolic rate adaptation, covarying for gender (men, women) if gender significantly added to the model. Linear and quadratic relation models were compared.

## RESULTS

**Fig. 2.**
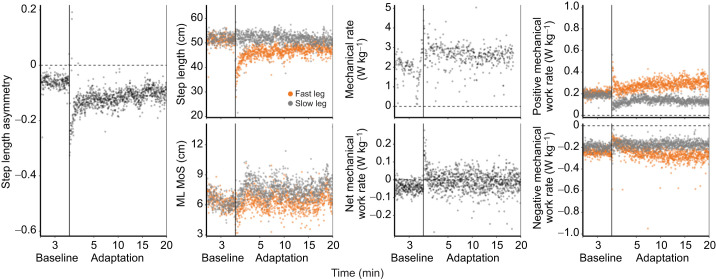
**Representative data from a single participant during baseline and adaptation.** Data are shown for: step length asymmetry, step length, mediolateral margin of stability (ML MoS), metabolic rate and mechanical work rates.

SLA and step lengths changed as hypothesized: the fast leg took longer steps resulting in a more-positive SLA over 20 min of SBT walking (Fig. 2). SLA for all participants is shown in Fig. S1. Table 2 provides the means±s.e.m. and significance levels for all adaptation coefficients. Although we observed significant reductions in positive and negative work rates, primarily by the fast leg (all timescales of adaptation *P*<0.001; Table 2), in agreement with previous studies (Sánchez et al., 2019, 2021; Selgrade et al., 2017), here we will focus on the aggregate measure of net work rate. Below, we present a summary of the key results.

**Table 2.**
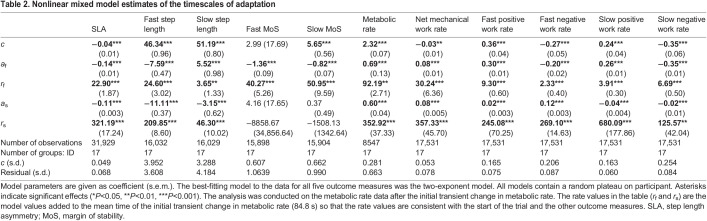
Nonlinear mixed model estimates of the timescales of adaptation

### Margins of stability adapt quickly, whereas energetic costs adapt gradually

**Fig. 3.**
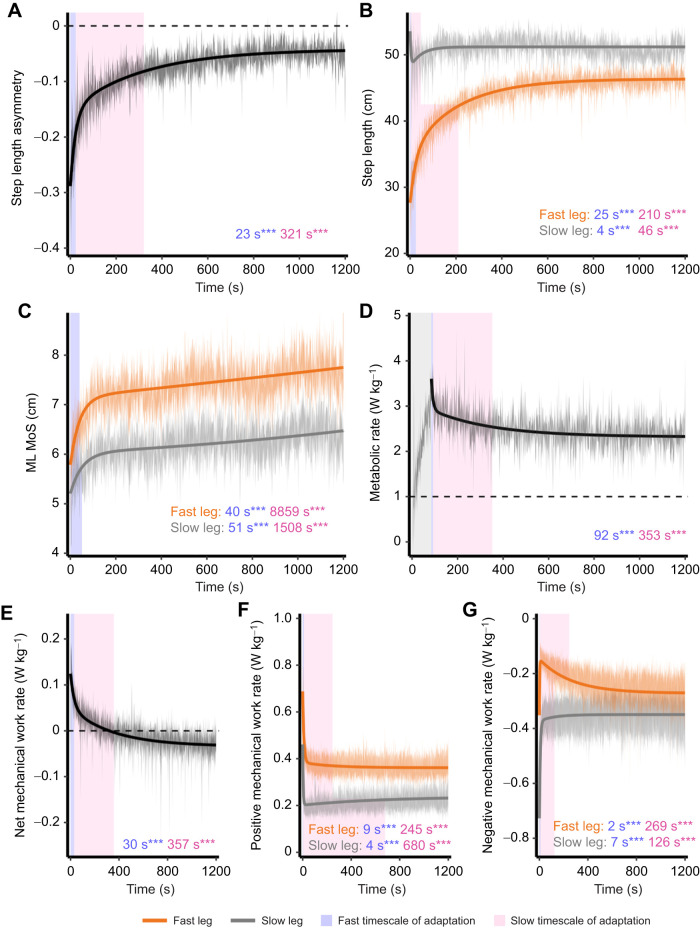
**Dual-rate models of gait adaptation.** Lines indicate the model-estimated adaptation and shaded ribbons indicate standard error of the actual data. Orange, fast leg; gray, slow leg. The numbers and shaded plot areas indicate the rates of adaptation of the fast timescale (blue) and the slow timescale (pink). (A) Step length asymmetry, (B) step length, (C) ML MoS, (D) metabolic rate (where the vertical gray shaded rectangle indicates the metabolic rate that has been removed from analyses), (E) net mechanical work rate, (F) positive mechanical work rate and (G) negative mechanical work rate. ***P<0.001.

The fast timescales of adaptation of SLA, fast-leg step length and slow-leg step length were significant (SLA: mean±s.e.m.=23±2 s, *P*<0.001; fast-leg step length: mean=25±3 s, *P*<0.001; slow-leg step length: mean=4±1 s, *P*=0.006; Fig. 3A,B). Additionally, the slow timescales of adaptation of SLA, fast-leg step length and slow-leg step length were also significant (SLA: mean=321±17 s, *P*<0.001; fast-leg step length: mean=210±9 s, *P*<0.001, Fig. 3B; slow-leg step length: mean=46±10 s, *P*<0.001; Fig. 3A,B).

ML MoS completed adaptation quickly, over a single, fast timescale (fast-leg: mean=40±5 s, *P*<0.001; slow-leg: mean=51±10 s, *P*<0.001; Fig. 3C). Conversely, both net metabolic rate (mean=353±37 s, *P*<0.001; Fig. 3D) and net mechanical work rate (mean=357±46 s, *P*<0.001; Fig. 3E) took longer to complete the slow timescale of adaptation.

### Stability predicts early SLA adaptation, while mechanical work predicts SLA adaptation over the entire trial

**Fig. 4.**
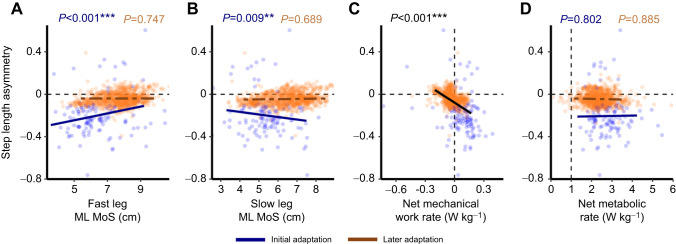
**Linear mixed regressions predicting step length asymmetry (SLA) during initial and later SLA adaptation.** Each data point represents the average over a binned 5-s interval for a participant, and data from all participants are shown. (A,B) SLA was predicted by both fast-leg and slow-leg ML MoS during initial but not later adaptation. Blue points, solid lines and *P*-values indicate the relationship between SLA and ML MoS during initial SLA adaptation, and orange points, dashed lines and *P*-values indicate the relationship between SLA and ML MoS during later SLA adaptation. (C) SLA was predicted by net mechanical work rate during the entirety of gait adaptation, regardless of adaptation phase. Blue points indicate initial adaptation, orange points indicate later adaptation, and the black line and *P*-value indicate the relationship between SLA and net mechanical work rate during the entirety of SLA adaptation. (D) SLA was not predicted by net metabolic rate during gait adaptation. Blue points, solid line and *P*-values indicate the relationship between SLA and net metabolic rate during initial SLA adaptation, and orange points, dashed line and *P*-values indicate the relationship between SLA and net metabolic rate during later SLA adaptation.

Fast-leg ML MoS was found to depend on the epoch (*F*_1,1074.3_=33.028, *P*<0.001) such that during initial adaptation a larger fast-leg ML MoS predicted a more positive SLA (*P*<0.001; Fig. 4A). Slow-leg ML MoS interacted with epoch (*F*_1,1069.4_=16.066, *P*<0.001) such that during initial adaptation, a smaller slow-leg ML MoS predicted a more positive SLA (*P*<0.001; Fig. 4B). During later adaptation, there was no significant relationship between ML MoS and SLA. Net mechanical work rate significantly predicted SLA during adaptation regardless of epoch such that a more positive SLA was predicted by a more negative net mechanical work rate (*F*_1,16.67_=13.599, *P*=0.002; Fig. 4C). Net metabolic rate (lagged behind SLA by 3 min) did not predict SLA during either epoch (Fig. 4D).

### Individuals with either poor or excellent aerobic fitness adapt gait to a greater extent than those with average aerobic fitness

**Fig. 5.**
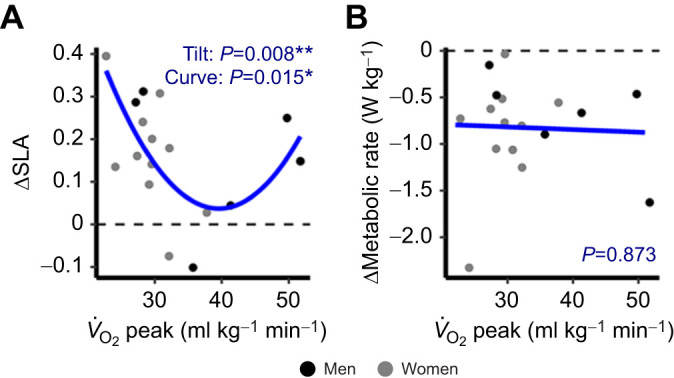
**The relationships between *V*_O_2__­ peak and adaptation of SLA and net metabolic rate.** (A) SLA and (B) net metabolic rate. Points reflect the change from the first 23 s of adaptation to the final 2 min of adaptation. Men are represented by black points, and women are represented by gray points, though gender did not affect the relationships between outcomes and *V*_O_2__ peak. Lines indicate the model-estimated relationships.

The relationships of *V*_O_2__ peak with SLA and metabolic rate adaptation are shown in Fig. 5. The best-fitting model to predict changes in SLA was a quadratic model with no effect of gender (Fig. 5A). There was a significant convexity (*B*=0.001, *P*=0.008) and a significant negative tilt (*B*=–0.018, *P*=0.015). The model *R*^2^ was 0.41. Predicted SLA adaptation from the start to the end of 20 min was minimal (+0.04) at a *V*_O_2__ peak of 39.4 ml kg^−1^ min^−1^, and SLA adaptation increased as *V*_O_2__ peak deviated in both directions. The best-fitting model to predict changes in metabolic rate was a linear model with no effect of gender (Fig. 5B). However, there was no evidence of a relationship between *V*_O_2__ peak and metabolic rate adaptation (*P*=0.873), and the model *R*^2^ was 0.001.

## DISCUSSION

This study determined the timescales of gait adaptation and mapped the relationships between step adaptation and stability, metabolic cost and mechanical cost adaptation. Stability adapted quickly and predicted SLA during the rapid initial phase of adaptation, emphasizing the role of stability in early gait adaptation. Conversely, mechanical and metabolic rates adapted more gradually, but only mechanical work rate predicted SLA during the later part of adaptation. Additionally, people with extreme aerobic fitness (high or low) adapted to a greater magnitude than those with average aerobic fitness. This study highlights the dynamic and shifting roles of stability and energetic cost during gait adaptation and presents novel findings on the influence of aerobic fitness on the extent of gait adaptation.

### Stability adapts rapidly and predicts early gait adaptation

Mediolateral stability underwent rapid adaptation, contrasting with the slower adjustments observed in mechanical and metabolic rates. The rapid stability adjustment aligns closely with the initial adaptation of SLA and the step length of the fast leg, reinforcing the idea that stability adaptation may play a pivotal role in driving initial gait adjustments consistent with previous studies (Park and Finley, 2022; Seethapathi et al., 2021). Further, during initial adaptation, the ML MoS of both the fast and slow legs predicted subsequent SLA, indicating that stability adaptation influences the early but not later stages of gait adjustment. Interestingly, a more positive SLA was associated with a larger fast-leg ML MoS but a smaller slow-leg ML MoS, suggesting that within the first ∼30 s of adaptation, shifting the CoM more over the slow leg contributes to a less negative SLA. This CoM shift not only occurs in initial gait adaptation, but persists during later adaptation; ML MoS remains larger for the fast leg and smaller for the slow leg, indicating that the CoM continues to stay more over the slow leg than the fast leg.

### Both metabolic and mechanical work adapt gradually, but only mechanical work predicts SLA during adaptation

The dual rates of step length adaptation replicate prior work (Darmohray et al., 2019; Mawase et al., 2013; Roemmich et al., 2016; Sánchez et al., 2021). These results also support prior findings that SLA is increased and net mechanical work rate reduced primarily by lengthening the fast-leg step length, whereas the slow-leg step length does not change considerably (Sánchez et al., 2021). SLA, net mechanical work rate and fast-leg mechanical work rate plateaued between 4 and 6 min, and metabolic rate plateaued in 5.9 min. Prior work has shown that net mechanical work rate is directly related to metabolic rate (Burdett et al., 1983; Donelan et al., 2002; Sánchez et al., 2019, 2021; Wanta et al., 1993), and the present study suggests that metabolic rate lags behind gait mechanics by 1.5 to 2.5 min during 3:1 belt-speed ratio gait adaptation. The decreases in mechanical and metabolic rates show that people make adjustments to reduce energy expenditure in a novel environment. However, although there was a strong relationship between a more negative net mechanical work rate and a more positive SLA, there was no evidence of a relationship between SLA and metabolic rate during initial or later adaptation. Although unexpected, these results are not surprising; Buurke et al. (2018) similarly did not find a relationship between the change in SLA and the change in metabolic power after 9 min of SBT walking. Also, although both metabolic rate and mechanical work involve some measurement error, the assumptions made in estimating metabolic rate using indirect calorimetry are broader, making it less tightly linked to the specific biomechanical adaptations being measured. It is possible that longer periods of gait adaptation such as 45 min (Sánchez et al., 2021), and therefore larger amounts of data per participant, are necessary to observe the relationship between metabolic power and adaptation.

It is important to note that the present study cannot dissociate stability from metabolic cost reduction. Mediolateral stability is a determinant of metabolic cost during typical walking and relates modestly to metabolic cost (Donelan et al., 2002, 2004). Stability optimization may contribute to reducing net mechanical work rate and metabolic rate, especially during initial adaptation. Future work should seek to determine the association between the rates of adaptation within individuals and the extent to which stability adaptation affects energetic adaptation.

### People at both ends of the aerobic fitness spectrum adapt to a greater extent than those with ‘average’ fitness

A subanalysis uncovered an intriguing link between aerobic fitness and SLA adaptation. Our previous research noted slower and less extensive gait adaptation in individuals with higher habitual exercise levels than those with lower levels (Brinkerhoff et al., 2023). We hypothesized that the latter group's greater adaptation might stem from poorer aerobic fitness, but we lacked metabolic data to confirm this. The present study provided a unique opportunity to explore the relationship between magnitudes of adaptation and aerobic fitness. We found a convex relationship between *V*_O_2__ peak and SLA: individuals with aerobic fitness below 36 ml kg^−1^ min^−1^ and above 43 ml kg^−1^ min^−1^ adapted to a greater extent within 20 min than those with aerobic capacities between 36 and 43 ml kg^−1^ min^−1^. Notably, the apex of this relationship aligned with fitness categories of ‘below average’ for men (37–41 ml kg^−1^ min^−1^) and ‘average’ for women (38–41ml kg^−1^ min^−1^) under the age of 25 (American College of Sports Medicine, 2019). However, our sample, limited to participants with less than 150 min per week of moderate–vigorous exercise, likely skewed towards lower aerobic fitness levels (Fig. 5). We concede that it also may be that the two participants with >45ml kg^−1^ min^−1^ *V*_O_2__ peak are driving the J-curve. Future research should explore this relationship in more aerobically heterogeneous samples, and in larger samples. These findings support the hypothesis that lower aerobic fitness drives adaptation to reduce relative energetic cost (Brinkerhoff et al., 2023); however, these findings also support the idea that higher aerobic fitness yields adeptness at reducing energetic cost, given that those who engage in more exercise can reach a minimum cost of transport during running while more sedentary adults do not (Cher et al., 2015). Individuals with below-average aerobic fitness adapt their gait to minimize energetic costs out of necessity, whereas those with above-average aerobic fitness do so out of capability.

Although our findings provide insight into how humans navigate novel environments, it is likely that some sensory feedback mechanisms influence the nervous system to produce the kinematic, kinetic and metabolic outputs observed in this study. However, from the current data, we cannot infer causal relationships between kinematics, kinetics and metabolic cost, nor can we fully disentangle the factors driving stability from those reducing energetic cost. It has long been thought that mediolateral dynamic balance is internally measured by the relative location of the projected CoM (i.e. center of gravity) to the CoP (Winter, 1995). Additionally, recent work suggests that the nervous system does not rely on the feedback from global blood-gas receptors to optimize energetic cost during walking (Wong et al., 2017). Instead, it might use local proprioceptive signals from Golgi tendon organs and spindles to estimate the demand from individual muscles and motor units, and to sense and adjust for specific muscle effort and potential fatigue, rather than for total-body energetic cost (Wong et al., 2017).

This study has several limitations. First, the sample studied were young adults and did not exercise regularly. Although a more homogeneous group improves statistical power and reduces the need for covariates, it also reduces generalizability to other populations. Future work should seek to understand the timescales of adaptation and the relationships between gait, stability and energetic cost in other populations. Second, we used a dual-rate model to measure gait adaptation. The results of this study and others (Brinkerhoff et al., 2023; Sánchez et al., 2021) suggest that a dual-rate model accurately represents multiple gait adaptation measures. Although our analysis focused on the dual-rate theory, future research should explore alternative models as new theoretical frameworks emerge.

Gait adaptation is a complex and multifaceted task consisting of multiple simultaneous processes. Stability adjusts rapidly during the initial phase of adaptation, whereas energetic costs, reflected in metabolic and mechanical rates, adapt gradually over time. Notably, stability predicts early gait adjustments, suggesting its pivotal role in driving initial changes. Our findings highlight a complex interplay between biomechanical and metabolic factors, with metabolic rate lagging behind gait mechanics during adaptation. Surprisingly, although there is a strong association between net mechanical work rate and SLA, no such relationship exists between metabolic rate and SLA. Our investigation also reveals a significant link between aerobic fitness and gait adaptation, with individuals at both ends of the fitness spectrum demonstrating greater adaptation than those with average fitness levels. These findings underscore the critical influence of biomechanical and metabolic factors in shaping gait adaptation strategies.

## Supplementary Material

10.1242/jexbio.249217_sup1 Supplementary information
