# Supplementary material for: The dual timescales of gait adaptation: initial stability adjustments followed by subsequent energetic cost adjustments
Source: J Exp Biol. 2024 Nov 28;227(23):jeb249217. doi: 10.1242/jeb.249217 (PMC11883409; doi:10.1242/jeb.249217)
Supplement: Supplementary information [file jexbio-227-249217-s1.pdf]

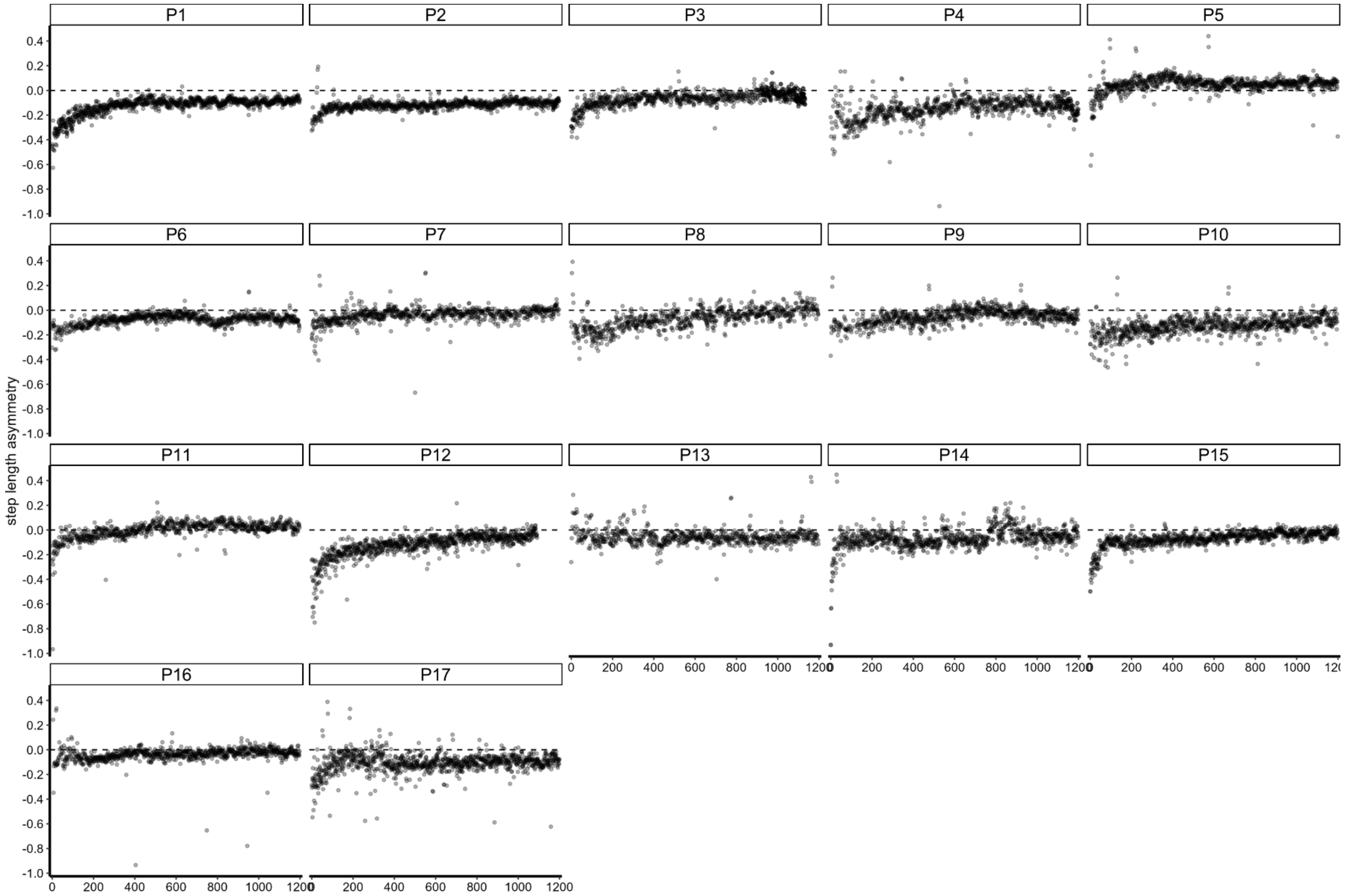

Fig. S1. Step length asymmetry over the entirety of 20 minutes of split-belt treadmill walking. The representative participant in Figure 2 was *P15* and the two participants that did not achieve a positive change in step length asymmetry from initial to alter adaptation were *P13* and *P16*.
